# Leiomyosarcoma Involving the Calcaneum With Delayed Gastric and Sacroiliac Lesions: A Case Report

**DOI:** 10.7759/cureus.116463

**Published:** 2026-09-17

**Authors:** Asmita Kaur, Rajat Gupta, Harpreet Singh, Gurkirat S Bhatti

**Affiliations:** 1 Gastroenterology, Liver and Digestive Diseases Centre, Amritsar, IND; 2 Orthopaedic Oncology, Bone Cancer Clinic, Panchkula, IND; 3 Radiology, Adesh Medical College and Hospital, Ambala, IND

**Keywords:** calcaneum, gastric lesion, leiomyosarcoma, metastasis, sacroiliac joint

## Abstract

Leiomyosarcoma involving the calcaneum is an uncommon presentation. A 63-year-old woman was evaluated in August 2023 for a right calcaneal lesion. Core needle biopsy demonstrated a high-grade sarcoma consistent with leiomyosarcoma involving the right calcaneum, with diffuse strong positivity for SMA, desmin, and h-Caldesmon and negativity for S100, CD34, MDM2, and SOX10*.* The MIB-1 labeling index was approximately 40%, with 6 to 8 mitoses per 10 high-power fields (HPF). The patient subsequently underwent total calcanectomy. Approximately two years and 10 months later, she developed an upper gastrointestinal bleed, and endoscopic evaluation identified a gastric lesion. Biopsy demonstrated a malignant mesenchymal tumor morphologically consistent with a metastatic tumor deposit in the setting of known leiomyosarcoma; detailed immunohistochemistry (IHC) studies were advised for confirmation and exact categorization but were not available. In June 2026, she developed swelling over the left sacroiliac region. Ultrasound-guided biopsy demonstrated a high-grade spindle cell sarcoma favoring leiomyosarcoma, with positivity for h-Caldesmon and desmin and a MIB-1 labeling index of approximately 60%. The patient is currently receiving systemic anticancer treatment, including pazopanib. This case highlights the unusual clinical course of leiomyosarcoma involving the calcaneum with subsequent gastric and sacroiliac lesions and emphasizes the importance of appropriate long term clinical and radiological follow-up.

## Introduction

Soft tissue sarcomas (STS) are rare malignant tumors, accounting for approximately 1% of adult cancers and comprising more than 70 molecular subtypes [1]. Leiomyosarcoma (LMS) is a malignant neoplasm showing smooth muscle differentiation and accounts for approximately 25% of diagnosed STS cases [2]. LMS can arise from smooth muscle cells or mesenchymal cells capable of smooth muscle differentiation and may occur at various anatomical sites, particularly the extremities and abdominopelvic organs [3]. LMS is associated with an unfavorable prognosis because of its potential for distant recurrence and reduced disease-free survival [4].

LMS involving the gastrointestinal tract is uncommon and represents a distinct entity from gastrointestinal stromal tumors (GISTs), which are more common mesenchymal neoplasms of the gastrointestinal tract. Although LMS and GIST may show overlapping morphological features, they have distinct molecular profiles and require different therapeutic approaches [5]. Historically, before the recognition of KIT and the improved molecular characterization of GISTs, some tumors diagnosed as gastrointestinal LMS were subsequently recognized as GISTs [6,7]. Immunohistochemistry (IHC) therefore plays an important role in distinguishing true smooth muscle tumors from GISTs and other mesenchymal neoplasms.

Leiomyosarcoma is characterized by malignant cells showing smooth muscle differentiation. Because several sarcomas and other mesenchymal tumors can have a spindle-cell appearance, IHC is an important part of diagnosis. Markers such as smooth muscle actin (SMA), desmin, and h-Caldesmon can support smooth muscle differentiation, whereas markers such as S100, SOX10, CD34, and MDM2 may help evaluate alternative diagnostic possibilities in the appropriate morphological context. The interpretation of these markers should be integrated with the tumor morphology, clinical history, and imaging findings rather than based on a single immunohistochemical marker [8].

Gastric involvement by LMS is particularly uncommon. Reported cases of gastric LMS have included both primary gastric tumors and metastatic deposits from LMS arising at other sites. Metastatic involvement of the stomach has been reported from leiomyosarcomas originating in the kidney, heart, uterus, and broad ligament [9-12]. Gastric metastasis may present with clinically significant gastrointestinal bleeding, including upper gastrointestinal bleeding reported in patients with metastatic uterine LMS [11]. These observations highlight the importance of considering metastatic LMS in patients with a history of LMS who subsequently develop a gastric lesion.

The present case describes leiomyosarcoma involving the right calcaneum, an unusual skeletal site, followed by a gastric malignant mesenchymal tumor morphologically consistent with a metastatic tumor deposit and subsequent involvement of the left sacroiliac region. The initial calcaneal tumor was treated with total calcanectomy, followed approximately two years and 10 months later by an upper gastrointestinal bleed associated with the gastric lesion and subsequent development of the left sacroiliac lesion. This unusual clinical course highlights the need to consider metastatic disease when new lesions develop at uncommon anatomical sites during long-term follow-up.

## Case presentation

Clinical timeline

The patient was first evaluated in August 2023 for a right calcaneal lesion. Radiographs were followed by MRI and 18F-fluorodeoxyglucose positron emission tomography/computed tomography (18F-FDG PET/CT), after which a core needle biopsy established a high-grade sarcoma consistent with leiomyosarcoma. She subsequently underwent total calcanectomy. Approximately two years and 10 months later, she developed an upper gastrointestinal bleed, and endoscopy identified a gastric lesion that was reported as a malignant mesenchymal tumor morphologically consistent with a metastatic tumor deposit in the setting of known leiomyosarcoma. In June 2026, she developed swelling over the left sacroiliac region, and biopsy demonstrated a high-grade spindle cell sarcoma favoring leiomyosarcoma.

Presentation and history

A 63-year-old woman was initially evaluated in August 2023 for a right calcaneal lesion. Her relevant past medical history included hysterectomy for uterine fibroids approximately 13 years earlier, in 2010. Core needle biopsy of the calcaneal lesion demonstrated a high-grade sarcoma consistent with leiomyosarcoma, with a Fédération Nationale des Centres de Lutte Contre le Cancer (FNCLCC) grade [13] of at least 2. She subsequently underwent total calcanectomy.

Preoperative workup

Initial radiographs of the right foot and ankle demonstrated an abnormal expansile lesion involving the calcaneum (Figure 1a-1b).

**Figure 1:**
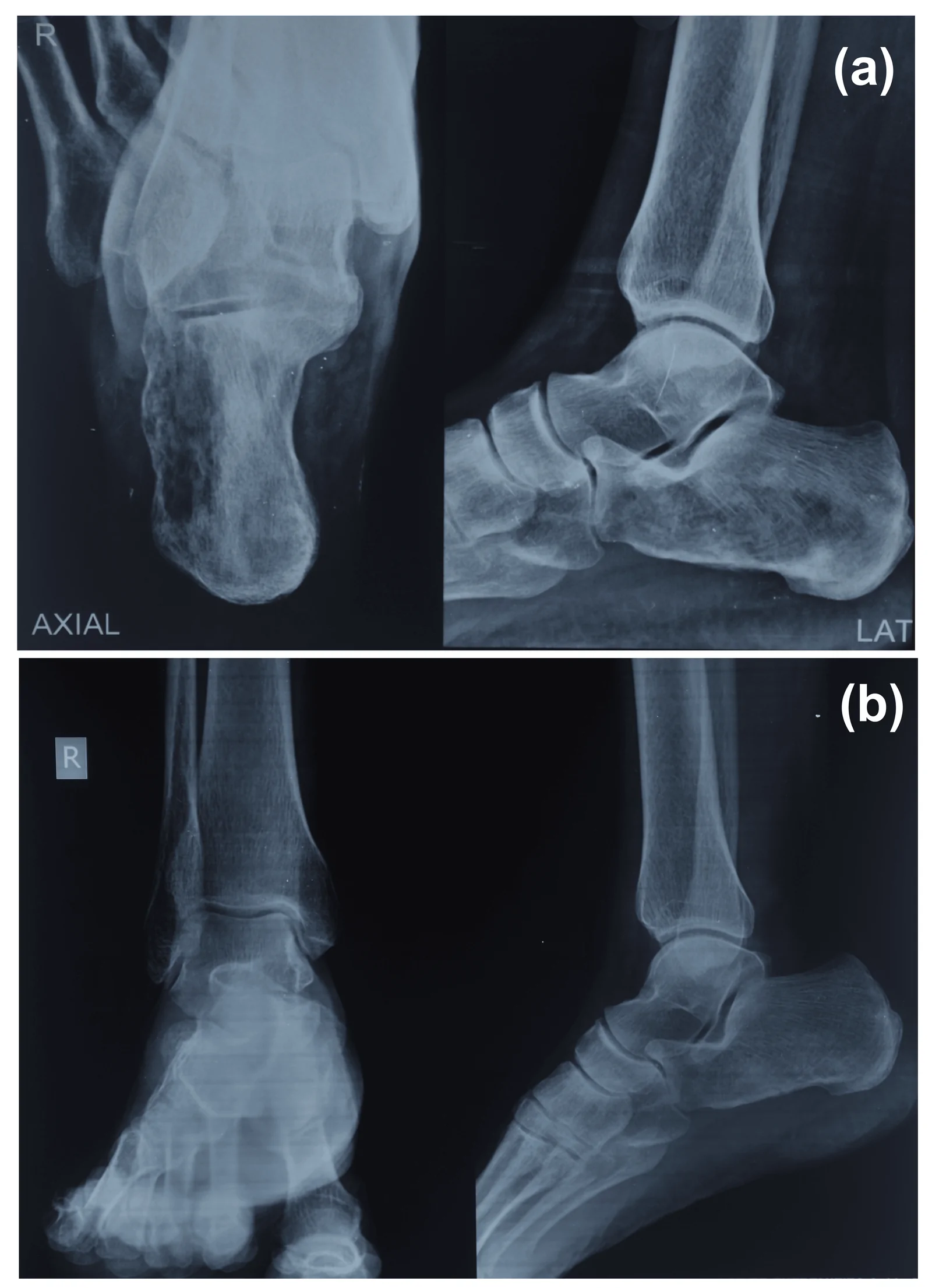
Preoperative radiographic evaluation of the right calcaneal lesion. (a) AP/axial and lateral radiographs showing an expansile destructive lesion of the right calcaneum. (b) Additional right ankle and foot views.

MRI was subsequently performed and demonstrated a well-defined intramedullary lesion involving most of the right calcaneum, measuring approximately 6.0 × 3.5 × 3.2 cm, with heterogeneous signal characteristics, cortical thinning, focal cortical breach, and adjacent soft-tissue extension (Figure 2a-2d).

**Figure 2:**
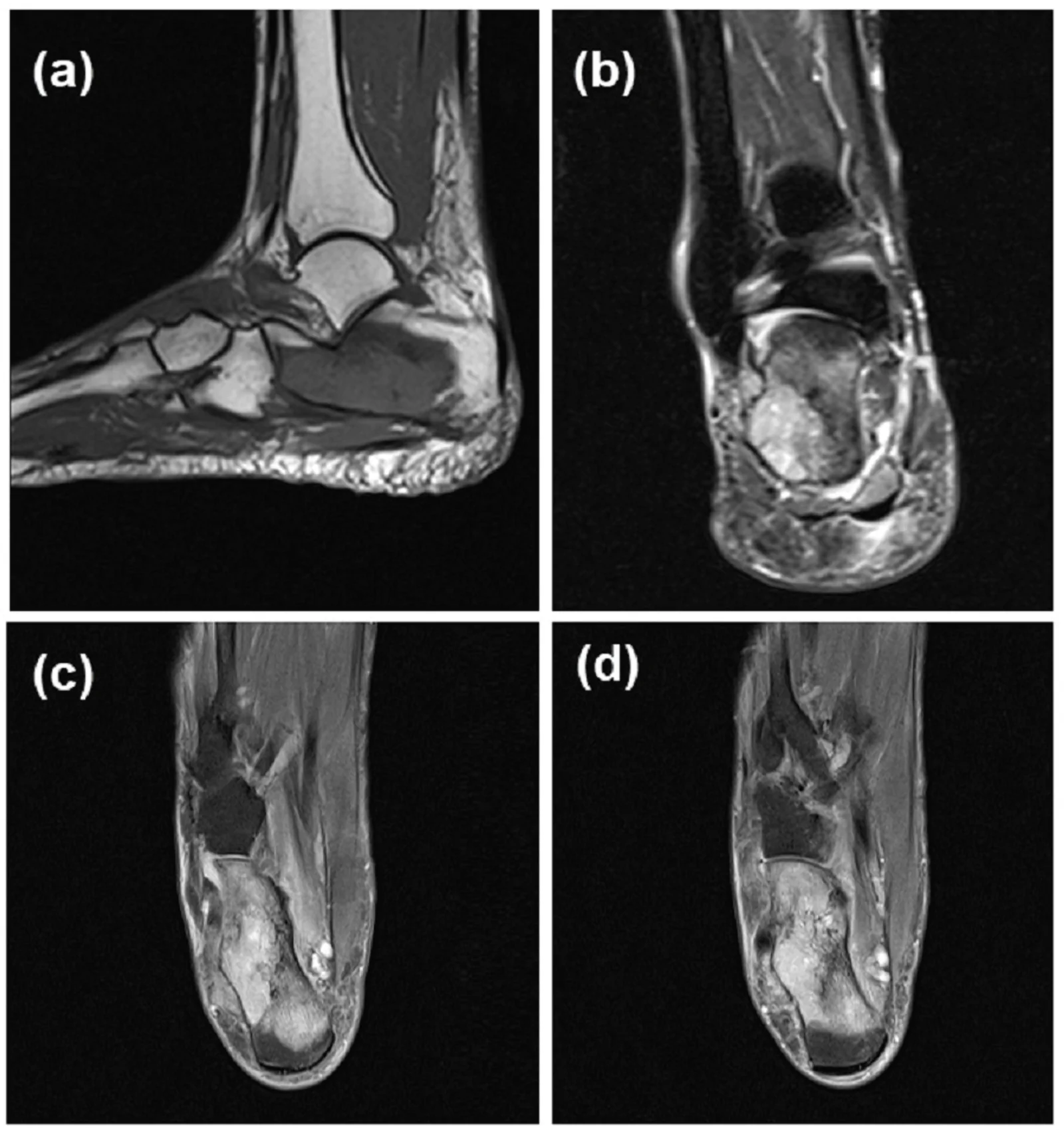
Preoperative MRI evaluation of the right calcaneal lesion. (a) Sagittal MRI showing the calcaneal lesion. (b) Coronal MRI showing heterogeneous signal abnormality. (c,d) Coronal images showing the lesion extent and adjacent soft-tissue abnormality.

The imaging appearance raised the possibility of a giant cell tumor; however, a malignant lesion, including sarcoma or metastatic disease, could not be excluded. An 18F-FDG PET/CT subsequently demonstrated increased FDG uptake in the right calcaneal lesion (Figure 3a-3d).

**Figure 3:**
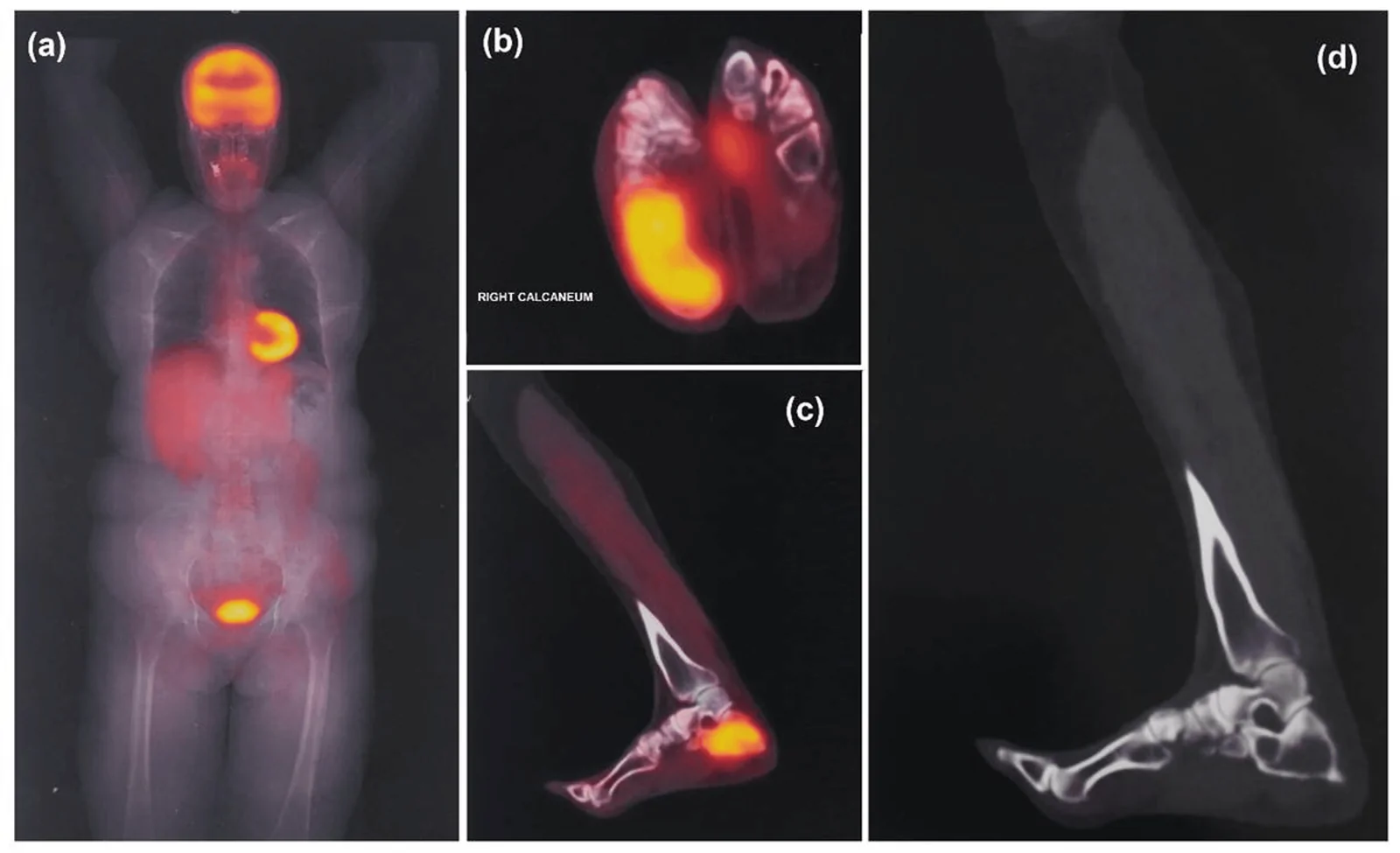
Preoperative 18F-FDG PET/CT evaluation of the right calcaneal lesion. (a) Whole-body PET/CT showing focal increased FDG uptake in the right calcaneal region. (b,c) Fused PET/CT images showing intense uptake corresponding to the lesion. (d) Sagittal CT showing the calcaneal abnormality. 18F-FDG PET/CT: 18F-fluorodeoxyglucose positron emission tomography/computed tomography.

The available records do not provide sufficient information to confirm the extent of staging performed at the time of the initial calcaneal diagnosis or whether other metastatic sites were identified. Because of the destructive osseous lesion and concern for malignancy, a core needle biopsy was performed and demonstrated a high-grade sarcoma consistent with leiomyosarcoma. The patient subsequently underwent total calcanectomy.

The biopsy demonstrated a high-grade sarcoma consistent with leiomyosarcoma involving the right calcaneum. IHC showed diffuse strong positivity for SMA, desmin, and h-Caldesmon, with negativity for S100, CD34, MDM2, and SOX10. The MIB-1 labeling index was approximately 40%, with 6 to 8 mitoses per 10 high-power fields (HPF), and the FNCLCC grade was at least grade 2. The pathology report specifically noted that the lesion could be considered a primary bone leiomyosarcoma only after excluding metastasis from another site and recommended clinicoradiological correlation.

Operative management

Following the diagnosis of high-grade leiomyosarcoma involving the right calcaneum, the patient underwent total calcanectomy as surgical treatment. The intraoperative photographs demonstrated the operative exposure and removal of the involved calcaneum (Figure 4a-4c), while the excised gross specimen and cut surface are shown in (Figure 4d-4f). Available records do not provide sufficient documentation regarding the specific surgical approach, intraoperative blood loss, margin status, reconstruction, or perioperative complications. Therefore, these details are not reported.

**Figure 4:**
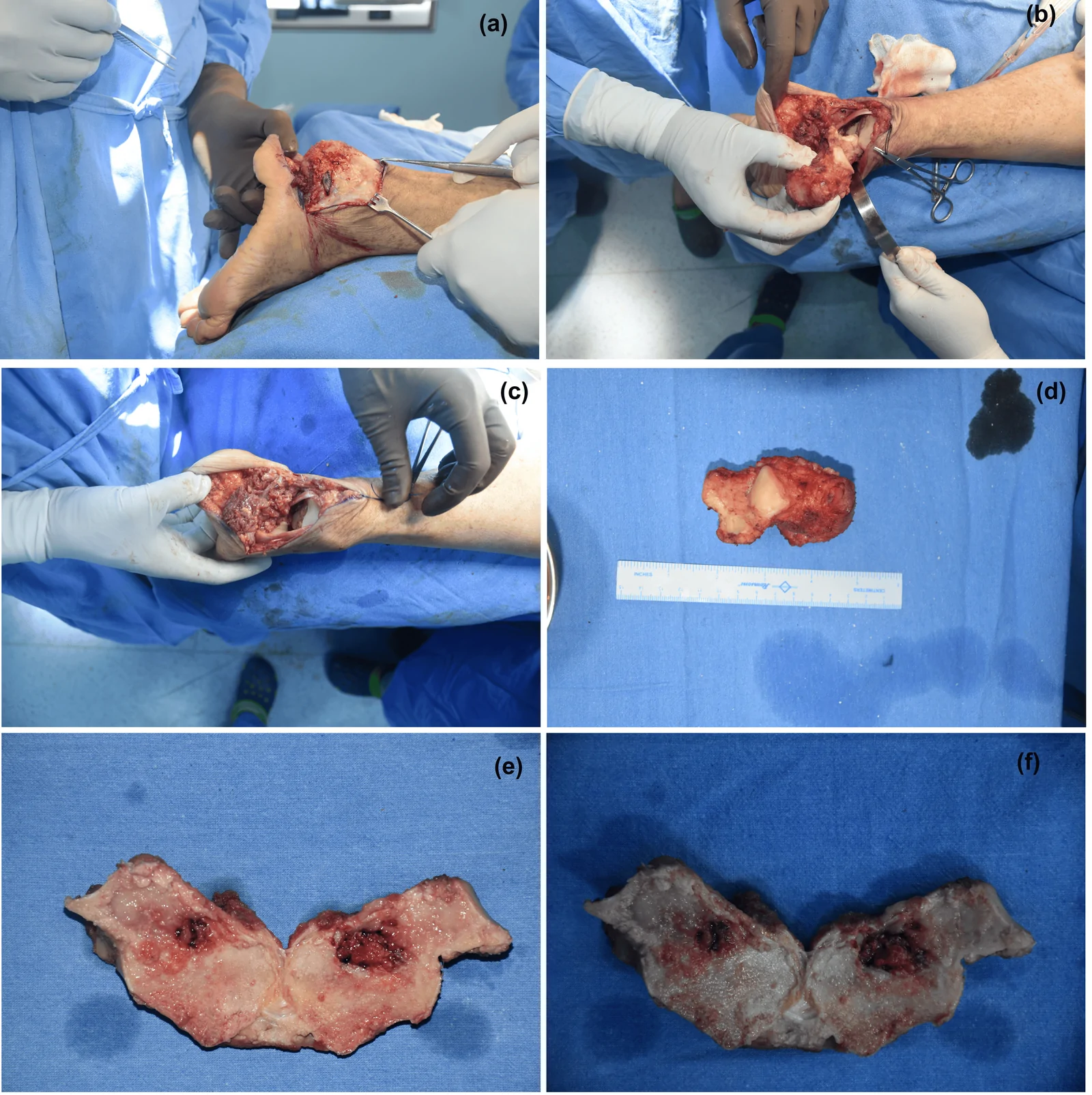
Intraoperative findings and gross specimen following total calcanectomy. (a-c) Intraoperative views of the right calcaneal lesion; (d) gross specimen; (e,f) cut surfaces of the resected specimen.

Histopathological and immunohistochemical examination

Histopathological evaluation of the right calcaneal lesion was performed on a core needle biopsy. Gross examination showed multiple tiny brown tissue fragments measuring 2.0 × 1.5 × 0.5 cm in aggregate, which were entirely processed in one paraffin block. Microscopic examination demonstrated a high-grade sarcoma consistent with leiomyosarcoma involving the right calcaneum. The tumor showed a MIB-1 labeling index of approximately 40% and a mitotic rate of 6 to 8 mitoses per 10 HPF. Necrosis and lymphovascular invasion were not identified in the submitted biopsy material. The tumor was assigned an FNCLCC grade of at least 2 (Table 1).

**Table 1:** Key pathological and immunohistochemical findings of the right calcaneal and left sacroiliac lesions. FNCLCC:  Fédération Nationale des Centres de Lutte Contre le Cancer; HPF: high-power field.

Lesion	Marker/finding	Result	Interpretation
Right calcaneal lesion	SMA	Diffuse strong positive	Supports the reported diagnosis of leiomyosarcoma
	Desmin	Diffuse strong positive	Supports the reported diagnosis of leiomyosarcoma
	h-Caldesmon	Diffuse strong positive	Supports the reported diagnosis of leiomyosarcoma
	S100	Negative	Negative marker in the reported IHC panel
	CD34	Negative	Negative marker in the reported IHC panel
	MDM2	Negative	Negative marker in the reported IHC panel
	SOX10	Negative	Negative marker in the reported IHC panel
	MIB 1	Approximately 40%	Reported proliferative index
	Mitotic rate	6 to 8/10 HPF	Reported mitotic activity
	FNCLCC grade	At least grade 2	High-grade sarcoma
Left sacroiliac lesion	h-Caldesmon	Positive	Supports the reported impression favoring leiomyosarcoma
	Desmin	Positive	Supports the reported impression favoring leiomyosarcoma
	CD68	Focal positive	Focal positivity reported
	CK	Negative	Negative marker in the reported IHC panel
	CD34	Negative	Negative marker in the reported IHC panel
	Myogenin	Negative	Negative marker in the reported IHC panel
	MyoD1	Negative	Negative marker in the reported IHC panel
	CKIT	Negative	Negative marker in the reported IHC panel
	MDM2	Negative	Negative marker in the reported IHC panel
	MIB-1	Approximately 60%	Reported proliferative index
	FNCLCC grade	At least grade 2	High-grade spindle cell sarcoma

Immunohistochemical examination demonstrated diffuse strong positivity for SMA, desmin, and h-Caldesmon, while the tumor cells were negative for S100, CD34, MDM2, and SOX10. Based on the histomorphological and immunohistochemical findings, the final pathological diagnosis was high-grade sarcoma consistent with leiomyosarcoma involving the right calcaneum, FNCLCC grade at least 2. The pathology report noted that the lesion could be considered primary bone leiomyosarcoma only after exclusion of metastasis from another site.

Histologically, the tumor was arranged in irregular fascicles and showed spindle to ovoid tumor cells with coarse chromatin, prominent nucleoli, eosinophilic cytoplasm, and frequent mitotic figures, including atypical mitoses (Figure 5a-5c). IHC demonstrated positivity for h-Caldesmon and desmin, with focal CD68 positivity, while CK, CD34, myogenin, MyoD1, CKIT, and MDM2 were negative (Figure 5d-5h).

**Figure 5:**
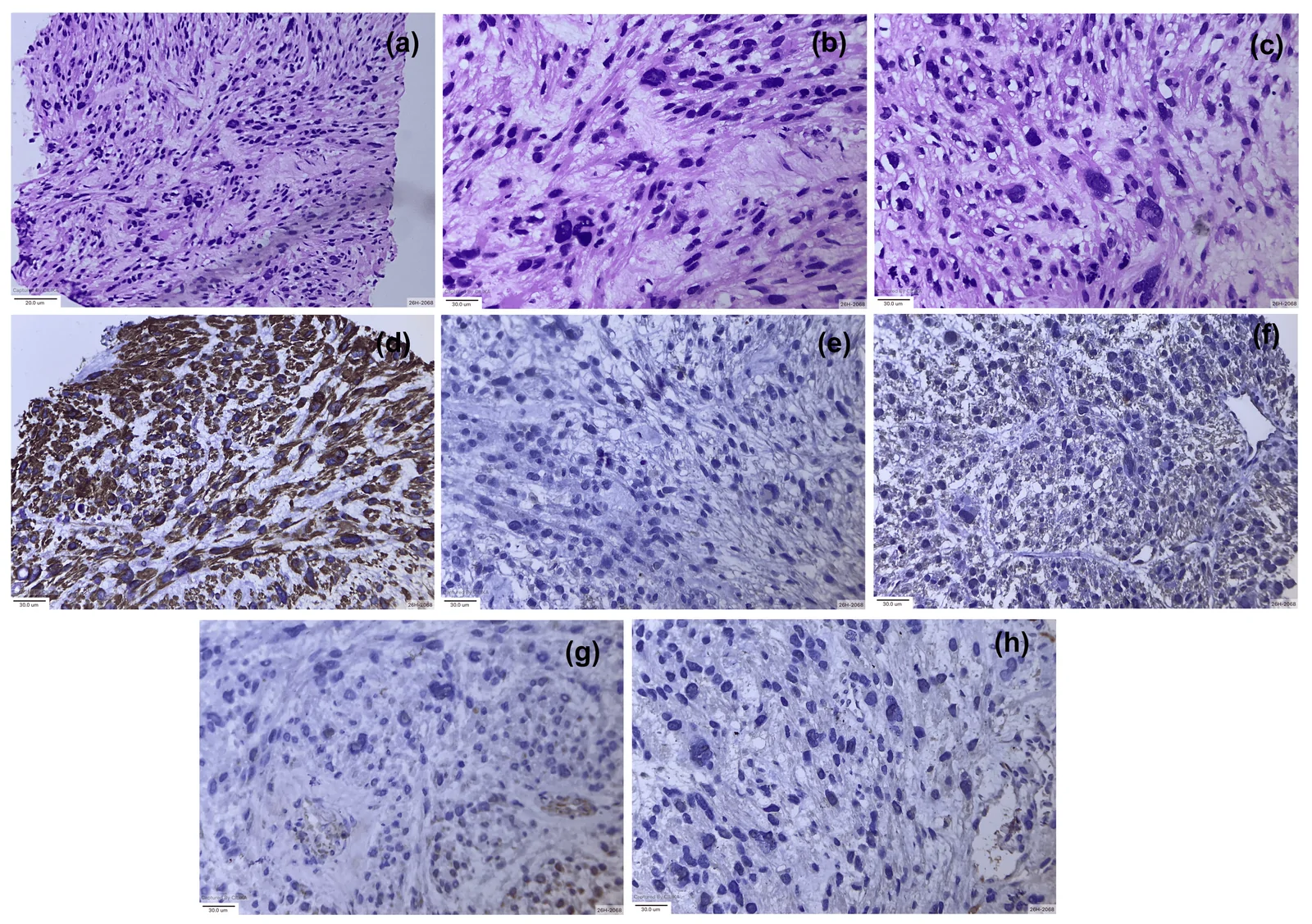
Histopathological and immunohistochemical findings of the left sacroiliac lesion. (a-c) Hematoxylin and eosin (H&E)-stained sections showing a high-grade spindle cell sarcoma (×40); (d) h-Caldesmon positivity (×40); (e-h) negative staining for CK, myogenin, MDM2, and CKIT, respectively (×40).

Histopathological evaluation of the gastric lesion

Histopathological evaluation of the gastric lesion was performed on endoscopic biopsy specimens obtained in June 2026. Gross examination of the labelled gastric lesion consisted of six filter paper-mounted biopsy fragments, each measuring approximately 0.2 cm. Microscopic examination showed gastric mucosa that was ulcerated in places, with expansion of the lamina propria by spindle-shaped cells demonstrating moderate nuclear pleomorphism and bizarre forms (Figure 6a-6b). The morphological features were interpreted as those of a malignant mesenchymal tumor morphologically consistent with a metastatic tumor deposit in the setting of known leiomyosarcoma. Biopsies obtained separately from the gastric antrum and corpus showed normal gastric mucosa and glands, with no evidence of *Helicobacter pylori* or neoplastic pathology. The final interpretation of the gastric lesion was malignant mesenchymal tumor, and detailed IHC studies were advised for confirmation and exact categorization but were not available.

**Figure 6:**
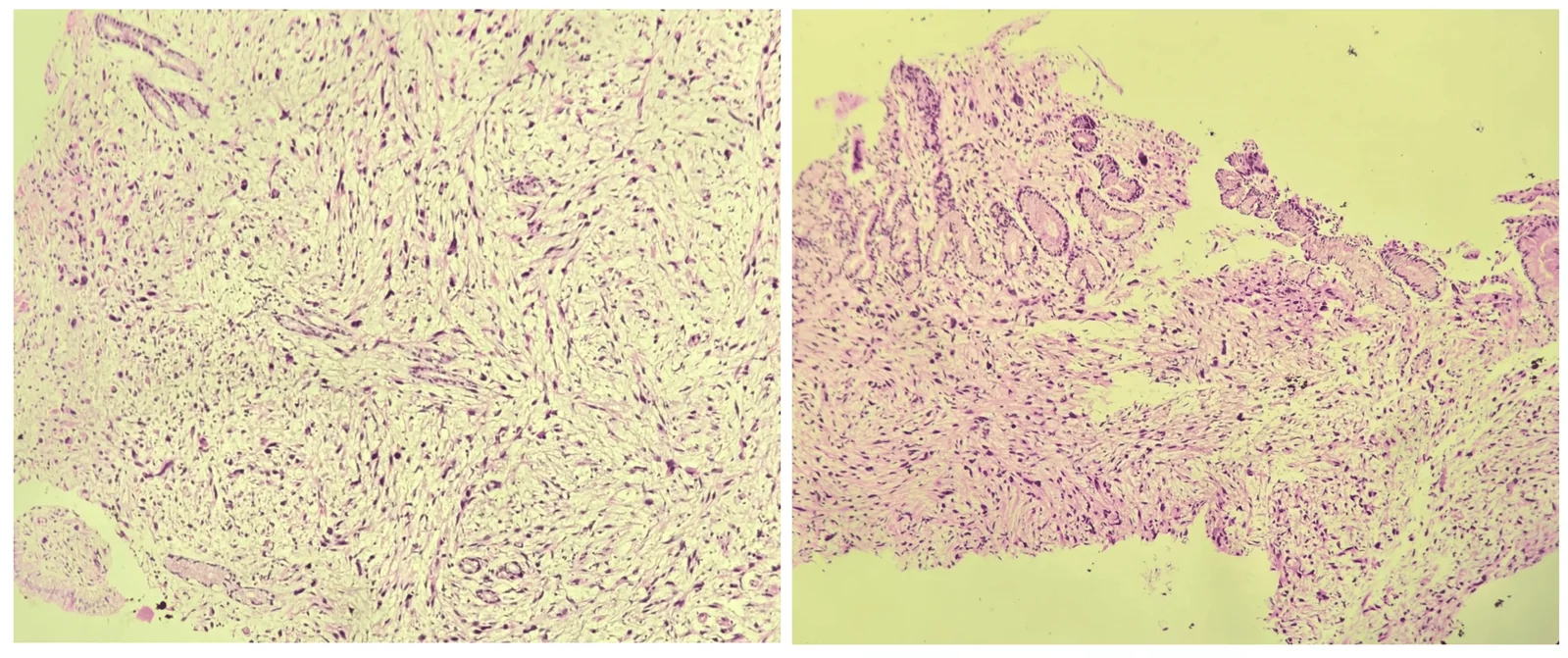
Histopathological findings of the gastric lesion. (a) Hematoxylin and eosin (H&E) section showing gastric mucosal ulceration and spindle-shaped tumor cells (×10); (b) spindle-shaped tumor cells with moderate nuclear pleomorphism and bizarre forms (×40).

Postoperative course and subsequent disease

Following total calcanectomy, the patient was followed clinically. Available records did not contain sufficient information regarding the specific postoperative imaging surveillance performed after total calcanectomy. Approximately two years and 10 months after the initial diagnosis in August 2023, she developed an upper gastrointestinal bleed. Endoscopic evaluation identified a gastric lesion, and biopsy demonstrated a malignant mesenchymal tumor morphologically consistent with a metastatic tumor deposit in the setting of known leiomyosarcoma. Detailed IHC studies were advised for confirmation and exact categorization but were not available. In 2026, the patient developed swelling over the left sacroiliac region. An ultrasound-guided biopsy of the lesion demonstrated a high-grade spindle cell sarcoma favoring leiomyosarcoma involving the SI joint. Histologically, the tumor was arranged in irregular fascicles and showed spindle to ovoid cells with coarse chromatin, prominent nucleoli, eosinophilic cytoplasm, and frequent mitotic figures, including atypical mitoses. IHC showed positivity for h-Caldesmon and desmin, with focal CD68 positivity, while CK, CD34, myogenin, MyoD1, CKIT, and MDM2 were negative. The MIB-1 labeling index was approximately 60%, and the tumor was assigned an FNCLCC grade of at least 2. The patient is currently receiving systemic anticancer treatment, including pazopanib. Objective treatment response data were not available at the time of reporting.

## Discussion

Leiomyosarcoma (LMS) is an aggressive sarcoma with a recognized propensity for distant metastasis. The lungs and liver are among the commonly reported metastatic sites, whereas gastric involvement is distinctly uncommon [14]. The present case is notable for an unusual clinical course beginning with LMS involving the right calcaneum, followed by development of a gastric malignant mesenchymal tumor approximately two years and 10 months later and subsequent involvement of the left sacroiliac region.

Gastric metastasis from LMS is rare, and only a limited number of cases have been reported. Uchiyama et al. described two patients with gastric metastasis from soft tissue LMS and highlighted the rarity of this metastatic pattern [14]. De Palma et al. reported a mean interval of 25.7 months between diagnosis of the primary tumor and development of gastric metastasis, with intervals ranging from 1 to 40 months [15]. In the present case, the gastric lesion developed approximately three years after the initial diagnosis, representing a relatively delayed development of a lesion considered morphologically consistent with metastatic disease. The clinical presentation was also comparable to previously reported cases. Gastrointestinal bleeding is among the recognized manifestations of gastric metastases, and Uchiyama et al. reported melena in one of their patients [14]. Similarly, Costa et al. described upper gastrointestinal bleeding caused by gastric metastasis from uterine LMS [11]. Our patient developed an upper gastrointestinal bleed that led to endoscopic identification and biopsy of the gastric lesion.

The gastric lesion was reported as a malignant mesenchymal tumor morphologically consistent with a metastatic tumor deposit in the setting of known leiomyosarcoma. However, definitive classification as metastatic leiomyosarcoma was not established because detailed IHC studies were advised for confirmation and exact categorization but were not available.

The diagnosis of gastric LMS requires careful distinction between primary gastric LMS and metastatic LMS. Kang et al. emphasized that, before the introduction of KIT immunohistochemistry, some tumors classified as gastric LMS were subsequently recognized as gastrointestinal stromal tumors (GISTs) [6]. True gastric LMS is therefore extremely rare, and immunohistochemistry is important for establishing smooth muscle differentiation and excluding GIST and other mesenchymal tumors. In their case, the tumor was positive for SMA and desmin and negative for CD117, DOG1, CD34, and S100 [6]. In our patient, the original calcaneal tumor demonstrated diffuse positivity for SMA, desmin, and h-Caldesmon, with negativity for S100, CD34, MDM2, and SOX10. The subsequent sacroiliac lesion similarly showed positivity for h-Caldesmon and desmin and was interpreted as a high-grade spindle cell sarcoma favoring LMS. The IHC findings in the original calcaneal lesion and subsequent sacroiliac lesion support the reported diagnoses in these two sites, while the gastric lesion remained morphologically consistent with a metastatic tumor deposit without independent gastric IHC confirmation.

The unusual skeletal site of the initial tumor is another important feature of this case. LMS involving the calcaneum is uncommon, and the subsequent development of gastric and sacroiliac lesions represents an unusual clinical distribution. Proposed routes of gastric metastasis include hematogenous, lymphatic, peritoneal, and direct spread [14,16]. The route of dissemination in our patient cannot be established from the available data. Therefore, although hematogenous dissemination is biologically plausible, it should not be considered definitive.

Management of gastric metastasis from LMS remains individualized because of its rarity. Uchiyama et al. suggested that local treatment may be considered in selected patients with symptomatic gastric metastases, particularly when bleeding affects quality of life [14]. Kang et al. noted that complete surgical resection is the standard approach for localized primary gastric LMS, but these principles cannot be directly applied to metastatic gastric disease [6]. In the present case, the patient developed lesions involving the stomach and left sacroiliac region and is currently receiving systemic anticancer treatment including pazopanib.

An additional consideration is the history of hysterectomy for uterine fibroids approximately 13 years before the calcaneal tumor was diagnosed. Because the original uterine histopathology is unavailable, a uterine origin cannot be definitively established or excluded. Accordingly, the tumor is appropriately described as LMS involving the calcaneum rather than definitively as a primary calcaneal LMS. Overall, this case highlights the potential for delayed and anatomically unusual metastatic progression of LMS and emphasizes the importance of considering gastric metastasis when patients with a history of LMS develop gastrointestinal bleeding. Long-term surveillance is important because clinically significant metastatic disease may become apparent several years after treatment of the initial tumor.

The main strength of this case is the unusual clinical course of leiomyosarcoma involving the calcaneum, followed by a gastric malignant mesenchymal tumor morphologically consistent with a metastatic deposit and subsequent sacroiliac involvement, with histopathological and immunohistochemical findings supporting leiomyosarcoma in the calcaneal and sacroiliac lesions. The availability of sequential imaging, operative photographs, and pathological findings provides comprehensive documentation of disease progression. However, this report has limitations. The histopathological report from the previous hysterectomy for uterine fibroids was unavailable, preventing definitive exclusion of a uterine origin. In addition, long-term treatment response and survival outcomes were unavailable, limiting assessment of prognosis and therapeutic effectiveness.

## Conclusions

This case highlights the unusual clinical course of leiomyosarcoma involving the calcaneum, followed by a gastric malignant mesenchymal tumor morphologically consistent with a metastatic deposit and subsequent involvement of the left sacroiliac region. Histopathological and immunohistochemical findings supported the diagnosis of leiomyosarcoma in the calcaneal lesion, while the sacroiliac lesion favored leiomyosarcoma. The case emphasizes the importance of considering metastatic disease when new gastrointestinal or skeletal lesions develop during follow-up in patients with leiomyosarcoma. Long-term clinical and radiological surveillance is important because disease progression may occur several years after initial treatment. The absence of the original uterine histopathology limits determination of whether the calcaneal lesion represented a primary bone tumor or metastatic disease from the uterus.

## References

[1] Ferrari A, Sultan I, Huang TT (2011). Soft tissue sarcoma across the age spectrum: a population-based study from the Surveillance Epidemiology and End Results database. Pediatr Blood Cancer.

[2] Guo X, Jo VY, Mills AM (2015). Clinically relevant molecular subtypes in leiomyosarcoma. Clin Cancer Res.

[3] George S, Serrano C, Hensley ML, Ray-Coquard I (2018). Soft tissue and uterine leiomyosarcoma. J Clin Oncol.

[4] Pisters PW, Leung DH, Woodruff J, Shi W, Brennan MF (1996). Analysis of prognostic factors in 1,041 patients with localized soft tissue sarcomas of the extremities. J Clin Oncol.

[5] Fletcher CD, Berman JJ, Corless C (2002). Diagnosis of gastrointestinal stromal tumors: a consensus approach. Hum Pathol.

[6] Kang WZ, Xue LY, Tian YT (2019). Leiomyosarcoma of the stomach: a case report. World J Clin Cases.

[7] Wang T, Zreik R, Leng B (2023). Primary gastric leiomyosarcoma: a rare case. Cureus.

[8] Choi JH (2026). Cutaneous soft tissue tumors in the 5th edition of the World Health Organization classification of skin tumors: key updates and new entities. J Pathol Transl Med.

[9] Yodonawa S, Ogawa I, Yoshida S (2012). Gastric metastasis from a primary renal leiomyosarcoma. Case Rep Gastroenterol.

[10] Abe K, Seo K, Yagi M, Sasajima Y, Yagi T, Inaba T, Fukushima R (2016). Gastrointestinal metastasis of cardiac leiomyosarcoma. Endoscopy.

[11] Costa M, Ivanova E, Esteves J (2016). Upper gastrointestinal bleeding due to gastric metastasis from a primary uterine leiomyosarcoma. Acta Clin Belg.

[12] Okagawa Y, Yoshinaga S, Noguchi E, Sekine S (2021). Gastric metastasis from primary leiomyosarcoma of the broad ligament. Jpn J Clin Oncol.

[13] Trojani M, Contesso G, Coindre JM (1984). Soft-tissue sarcomas of adults; study of pathological prognostic variables and definition of a histopathological grading system. Int J Cancer.

[14] Uchiyama T, Nakamura T, Nakata K, Adachi R, Hagi T, Asanuma K, Sudo A (2023). Gastric metastasis in patients with leiomyosarcoma: a case report. Biomed Rep.

[15] De Palma GD, Masone S, Rega M (2006). Metastatic tumors to the stomach: clinical and endoscopic features. World J Gastroenterol.

[16] Namikawa T, Hanazaki K (2014). Clinicopathological features and treatment outcomes of metastatic tumors in the stomach. Surg Today.

